# Acute abdominal pain due to sigmoid volvulus with persistent descending mesocolon: a case report

**DOI:** 10.1186/s13256-022-03598-y

**Published:** 2022-10-01

**Authors:** Hirotaka Kato, Hiroyuki Kinoshita, Yoshifumi Sakata

**Affiliations:** Department of Surgery, Saiseikai Wakayama Hospital, 45 juniban-cho, Wakayama, Wakayama 640-8158 Japan

**Keywords:** Persistent descending mesocolon, Anomaly, Symptomatic, Sigmoid volvulus, Case report

## Abstract

**Background:**

Persistent descending mesocolon, an anomaly of fixation of the mesentery of the descending colon, can sometimes cause complications such as intestinal obstruction and intussusception. We present the first reported case of sigmoid volvulus with persistent descending mesocolon.

**Case presentation:**

An 82-year-old Japanese man had intermittent lower abdominal pain. Abdominal computed tomography showed dilation and a shift to the right side of the sigmoid colon, but no findings of volvulus. The next day, he presented continuous lower abdominal pain with bloody stool. A second abdominal computed tomography showed strangulation and dilation of the sigmoid colon, with shift from the right side of the abdominal cavity to the pelvic space. This suggested the descending colon was running to the medial side with sigmoid volvulus. Emergency surgery was performed for volvulus with persistent descending mesocolon. Operative findings revealed dilation of the sigmoid colon with a partial poorly colored region and strangulation that caused volvulus. After releasing the strangulation of the sigmoid colon, the descending colon was revealed to be running more to the medial side, with adherence to small intestinal mesentery. There was no Toldt’s fusion fascia at the descending colon. Persistent descending mesocolon was therefore diagnosed due to abnormality of fixation of the descending colon. The sigmoid colon, including the poorly colored region, was resected and reconstructed, while the inferior mesenteric and left colonic arteries were preserved because of the complexity of the vascular system running around the descending and sigmoid colon due to the shortened mesentery. These findings were pathologically compatible with circulatory compromise and intestinal degeneration due to sigmoid volvulus. The patient had no complications after discharge, including in relation to defecation.

**Conclusion:**

Persistent descending mesocolon can occasionally cause acute abdominal symptoms requiring immediate treatment. A computed tomography finding of the descending colon running more to the medial side than ordinary cases can aid diagnosis of persistent descending mesocolon.

## Background

Persistent descending mesocolon (PDM) is defined as an anomaly of fixation of the mesentery of the descending colon, where there has been a shift of the descending colon to a more medial position [1]. PDM is usually asymptomatic, but there is growing awareness because of the increasing morbidity of colorectal cancer, the prevalence of laparoscopic surgery [2], and the difficulty of surgical procedures due to the complexity of the vascular system. There have only been a few reports of complications with PDM, specifically on intestinal obstruction [3] and intussusception [4]. We herein report a rare case of sigmoid volvulus with PDM with presentation of abdominal pain.

## Case presentation

**Fig. 1:**
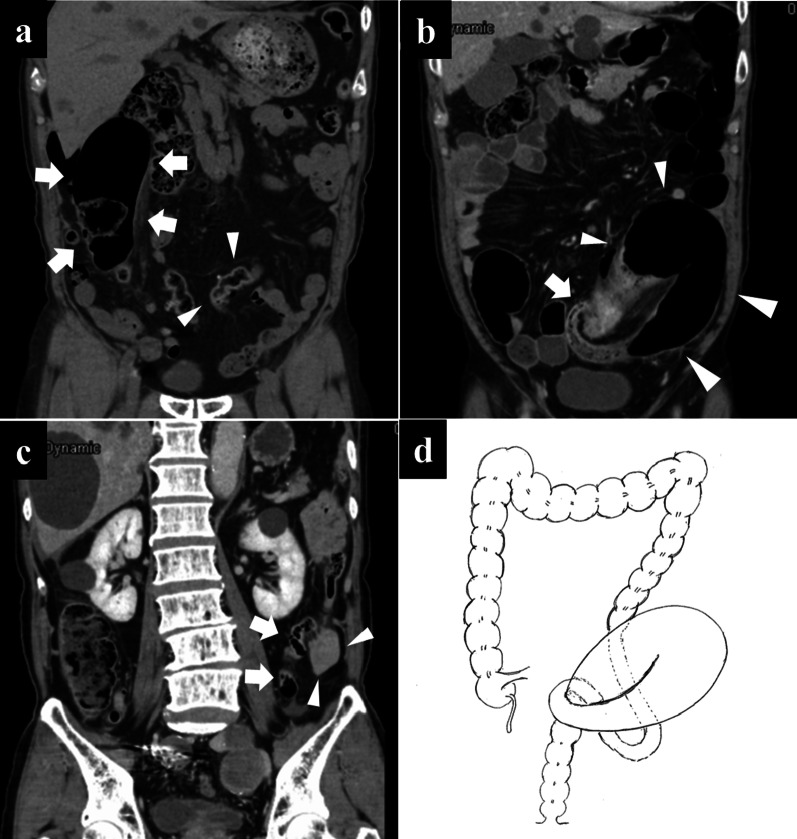
First abdominal CT shows dilated sigmoid colon on the right side (arrows) and shifted descending colon towards the medial side (arrowheads) (**a**). Second abdominal CT shows strangulation (arrows) and the oral dilated sigmoid colon (arrowheads) (**b**). Second abdominal CT also shows descending colon shifted to median side (arrows) and small intestine located at the lateral side of the descending colon (arrowheads) (**c**). The patient presented sigmoid volvulus with PDM (**d**)

An 82-year-old Japanese man visited hospital with intermittent lower abdominal pain. He had no history of admission or diagnosis related to abdominal pain. Abdominal computed tomography (CT) showed shift of the descending and sigmoid colon toward the right side with dilation (Fig. 1a), but there were no significant findings suggestive of the reason for acute abdominal pain. The patient revisited hospital the next day with continuous lower abdominal pain and bloody stool. Vital signs were oxygen saturation 97% under room air, respiratory rate 18 breaths/minute, pulse 106 beats/minute, blood pressure 173/97 mmHg, and body temperature 37.6 °C. Physical examination revealed abdominal distension and tenderness. Blood examination showed white blood cell (WBC) 11320/μL, C-reactive protein (CRP) 3.81 mg/dL, and hemoglobin (Hb) 8.7 g/dL. Blood gas analysis showed pH 7.467, pO_2_ 94.2 mmHg, pCO_2_ 34.9 mmHg, HCO_3_ 24.6 mmol/L, base excess (BE) 1.1 mmol/L, and lactate (Lac) 0.7 mmol/L (Table 1). Secondary abdominal CT showed strangulation and dilation of the sigmoid colon in the pelvic space that suggested sigmoid volvulus, although there was no perceptible free air or ascites (Fig. 1b). The descending colon was running more to the medial side, similar to in the first CT image (Fig. 1c). The patient underwent endoscopic reduction for sigmoid volvulus with PDM (Fig. 1d). Endoscopic reduction failed to release the volvulus, however, and emergency surgery was performed.

**Table 1:** preoperative blood examination and blood gas analysis

*Blood examination*			CRP	3.81	mg/dL
WBC	11320	/μL	T-Bil	0.8	mg/dL
RBC	395	10^4^/μL	D-Bil	0.1	mg/dL
Hb	8.7	g/dL	CEA	4.2	ng/dL
Ht	29.1	%	CA19-9	34.5	U/mL
PLT	45.8	10^4^/μL	Glu	120	mg/dL
PT	90.7	%	HbA1c	5.4	%
PT-INR	1.05				
APTT	38.2	Seconds	*Blood gas analysis*		
TP	7.1	g/dL	pH	7.467	
Alb	3.8	g/dL	pCO_2_	34.9	mmHg
CK	131	IU/L	pO_2_	94.2	mmHg
AST	24	IU/L	HCO_3_	24.6	mmol/L
ALT	26	IU/L	BE	1.1	mmol/L
LDH	213	IU/L	Hb	8.4	g/dL
ALP	26	IU/L	Ht	25	%
γGTP	27	IU/L	Na	134	mEq/L
AMY	49	IU/L	K	4.2	mEq/L
Lip	17	IU/L	Cl	103	mEq/L
Cre	1.1	mg/dL	Ca	1.02	mmol/L
BUN	16.6	mg/dL	Glu	114	mg/dL
eGFR	49.3	mL/minute	Lac	0.7	mmol/L
Na	138	mEq/L			
K	4.3	mEq/L			
Cl	104	mEq/L			

Alb, albumin; ALP, alkaline phosphatase; ALT, alanine aminotransferase; AMY, amylase; APTT, activated partial thromboplastin time; AST, aspartate aminotransferase; BE, base excess; BUN, urea nitrogen; Ca, calcium; CA19-9, carbohydrate antigen 19-9; CEA, carcinoembryonic antigen; CK, creatine kinase; Cl, chloride; Cre, creatinine; CRP, C-reactive protein; D-Bil, direct bilirubin; eGFR, estimated glomerular filtration rate; Glu, glucose; γGTP, gamma glutamyl transpeptidase; Hb, hemoglobin; HbA1c, hemoglobin A1c; HCO_3_, bicarbonate; Ht, hematocrit; K, potassium; Lac, lactate; LDH, lactic dehydrogenase; Lip, lipase; Na, natrium; PLT, platelet; pCO_2_, partial pressure of carbon dioxide; pO_2_, partial pressure of oxygen; PT, prothrombin time; PT-INR, prothrombin time international ratio; RBC, red blood cell; T-Bil, total bilirubin; TP, total protein; WBC, white blood cell

### Operative findings

**Fig. 2:**
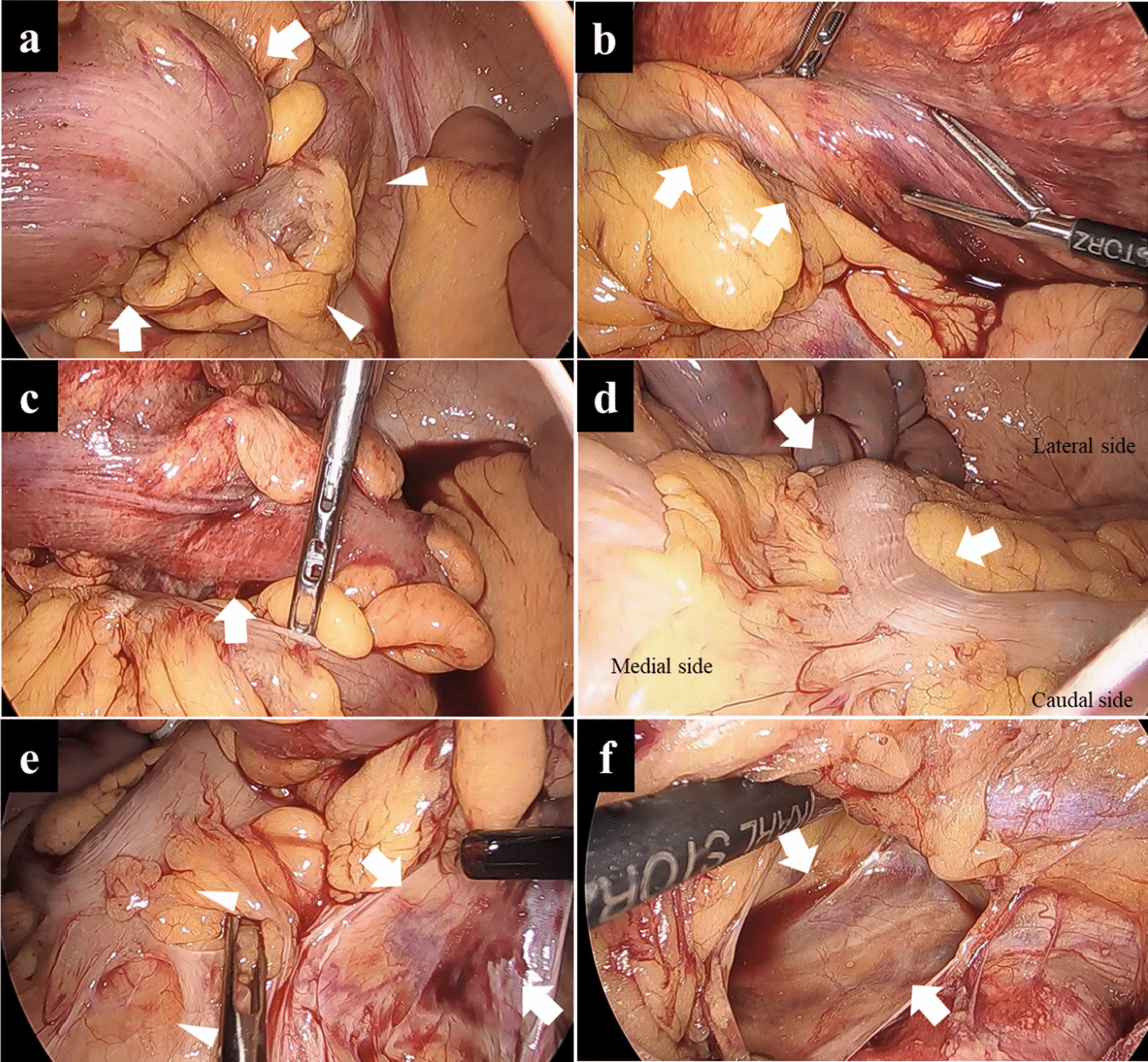
Operative findings revealed dilated sigmoid colon (arrows) and strangulation of the sigmoid colon (arrowheads) (**a**). There was twist of sigmoid colon and mesentery (arrows) (**b**). After release of the volvulus (arrows) (**c**), the descending colon ran more to the medial side with adhesion to the intestinal mesentery (arrows) (**d**), and the sigmoid colon had shortened mesentery (arrows) and adhesion to the small colon mesentery (arrowheads) (**e**). There was no Toldt’s fusion fascia in the medial approach (arrows) (**f**)

The procedure was performed laparoscopically. Dilated sigmoid colon with partial poorly colored region was seen within the pelvic cavity (Fig. 2a). Strangulation of the sigmoid colon was detected (Fig. 2a, b) and released as soon as possible (Fig. 2c). The descending colon was running more to the medial side with adherence to small intestinal mesentery (Fig. 2d), suggesting PDM. The sigmoid colon had shortened mesentery (Fig. 2e). Detachment of the adhesion between the descending colon and small intestinal mesentery, dissection of the sigmoid colonic mesentery toward the root of inferior mesenteric artery (IMA), and identification of the root of the IMA, were by medial approach. There was no Toldt’s fusion fascia at the descending colon, so the abdominal cavity at the lateral side of the descending colon was reached directly (Fig. 2f). PDM was therefore diagnosed due to abnormality of fixation of the descending colon. Detaching the partial adhesion between the colon and the abdominal wall, we dissected the sigmoid colon while preserving the superior rectal artery (SRA). Elongating the wound site and pulling out the sigmoid colon, we resected the sigmoid colon including the poorly colored segment. The left colonic artery (LCA) was not dissected because there was a possibility of colonic ischemia due to shortened sigmoid mesentery. Colon–colon anastomosis was made by circular stapler. Inserting the drain into the pelvic bottom and washing the abdominal cavity, we finished the operation. The operation time was 182 minutes and the amount of blood loss was 108 mL.

Pathological findings were compatible with circulatory compromise and intestinal degeneration due to sigmoid volvulus. Postoperatively, the patient had no complications after being discharged on postoperative day 8, including in relation to defecation.

## Discussion

PDM is an anomaly of fixation of the descending colonic mesentery. The descending colon is located toward the medial side and the sigmoid colon is at the right side of the abdomen [1]. The descending colon does not adhere to the parietal peritoneum and the left colon adheres to the small intestinal mesentery [5]. The descending mesocolon is normally fused and fixed to the posterior and lateral parietal peritoneum in the fifth month of gestation, but the primitive dorsal mesocolon may fail to fuse with the parietal peritoneum [6]. The descending colon therefore has mobility and variation in its position in PDM. The PDM is classified into three types according to the degree of the displacement of the descending colon and the range of the adhesion. Type A is the complete lack of fixation of both the ascending and descending colon, along with the absence of the transverse colon. Type B is moderate displacement of the descending colon to the midline or slightly to the left of the midline. Type C is the marked displacement of the descending colon with paracecal fixation [1]. PDM is also categorized into long-S and short-S types according to the length of the sigmoid colon and adhesion to the descending colon. Long-S type has excessive adhesion between the long sigmoid colon and the descending colon. In the short-S type, the descending colon runs straight without any adhesion to the sigmoid colon [6]. In the present case, the first abdominal CT showed that the descending colon had shifted toward the medial side and the sigmoid colon shifted toward the right side, suggesting type B PDM. Abdominal CT finding of the sigmoid colon being too long and meandering was also suggestive of long-type PDM.

PDM is usually asymptomatic [6], but has been known to cause primary intestinal obstruction [2], intussusception [3], and colonic volvulus at the descending colon [7]. PDM has recently come to be recognized, along with the increasing prevalence of colorectal cancer and the spread of laparoscopic and robotic surgery for colorectal cancer [8, 9]. PDM has been observed in 2.3% of cases of left colorectal cancer [10]. However, it is little known outside of the colorectal cancer field because it is usually asymptomatic and the detection rate has been reported to be as little as 8.9% [11], so it requires wider understanding.

Volvulus is caused by twisting of the intestine on its mesenteric axis and can result in intestinal obstruction. It can occur in the sigmoid, cecum, and transverse colon that possess mesentery. Volvulus can sometimes reduce spontaneously with chronic recurrence, but can occur acutely with greater frequency. Predisposing factors to volvulus formation include presence of mesocolon in the descending colon and a very long colon [12]. PDM can therefore contribute to colonic mobility and cause volvulus at the descending or sigmoid colon. However, sigmoid volvulus with PDM has been rarely reported because the sigmoid colon is extensively adhered to the abdominal wall and the pelvic cavity in PDM and is usually unlikely to cause volvulus at the sigmoid colon.

Characteristic CT findings for PDM are a shift of the descending colon to the medial side and shift of the sigmoid colon to right side, as well as the descending colon artery, sigmoid colon artery, and superior rectal artery often branching radially from the IMA [5, 9]. Characteristic operative findings for PDM include no Toldt’s fusion fascia, shortened colonic mesentery, and adhesion between the descending colon and small intestinal mesentery [13]. Moreover, the LCA and the inferior mesenteric vein (IMV) often run closely to the marginal artery because of the shortening of the mesentery. PDM is often therefore diagnosed intraoperatively. In the present case, first and second abdominal CT showed mobilization and volvulus of the sigmoid colon within a short time. In intraoperative findings, adhering to the abdominal wall to a small degree, the long sigmoid colon had much mobility and caused volvulus. The descending colon had shifted towards the median side and adhered to the small intestinal mesentery after the sigmoid colon was released from volvulus. PDM was therefore suspected intraoperatively. Medial approach was performed to resect poorly colored sigmoid colon but no Toldt’s fusion fascia was revealed, so PDM was diagnosed intraoperatively. Considering the possibility of postoperative complications such as anastomotic failure or anastomotic stenosis due to ischemia of the anastomosis site [14], we performed a procedure to preserve the IMA, LCA, and SRA. Postoperative follow-up for defecation was therefore required.

## Conclusion

PDM can occasionally cause acute abdominal symptoms that require immediate treatment. A CT finding of descending colon running more to the medial side of the abdominal cavity compared with ordinary cases can aid diagnosis of PDM.

## Data Availability

All laboratory data are available from the Department of Surgery, Saiseikai Wakayama Hospital, Wakayama, Japan, upon reasonable request.

## Abbreviations

BE: Base excess
CRP: C-reactive protein
CT: Computed tomography
IMA: Inferior mesenteric artery
Lac: Lactate
LCA: Left colic artery
PDM: Persistent descending mesocolon
SRA: Superior rectal artery
WBC: White blood cell
